# Genome-Wide Identification of *G3BP* Family in U’s Triangle *Brassica* Species and Analysis of Its Expression in *B. napus*

**DOI:** 10.3390/plants14142247

**Published:** 2025-07-21

**Authors:** Alain Tseke Inkabanga, Qiheng Zhang, Shanshan Wang, Yanni Li, Jingyi Chen, Li Huang, Xiang Li, Zihan Deng, Xiao Yang, Mengxin Luo, Lingxia Peng, Keran Ren, Yourong Chai, Yufei Xue

**Affiliations:** 1 Integrative Science Center of Germplasm Creation in Western China (Chongqing) Science City, College of Agronomy and Biotechnology, Southwest University, Chongqing 400715, China; alain1982@email.swu.edu.cn (A.T.I.); zqh906190@email.swu.edu.cn (Q.Z.); 2 Engineering Research Center of South Upland Agriculture, Ministry of Education, Academy of Agricultural Sciences, Southwest University, Chongqing 400715, China

**Keywords:** G3BP, *Brassica napus*, seed quality traits, abiotic/biotic stresses, *Brassica* species, genome-wide analysis, expression analysis

## Abstract

The RasGAP SH3 domain binding protein (G3BP) is a highly conserved family of proteins in eukaryotic organisms that coordinates signal transduction and post-transcriptional gene regulation and functions in the formation of stress granules. G3BPs have important roles in abiotic/biotic stresses in mammals, and recent research suggests that they have similar functions in higher plants. *Brassica* contains many important oilseeds, vegetables, and ornamental plants, but there are no reports on the *G3BP* family in *Brassica* species. In this study, we identified *G3BP* family genes from six species of the U’s triangle (*B. rapa*, *B. oleracea*, *B. nigra*, *B. napus*, *B. juncea*, and *B. carinata*) at the genome-wide level. We then analyzed their gene structure, protein motifs, gene duplication type, phylogeny, subcellular localization, SSR loci, and upstream miRNAs. Based on transcriptome data, we analyzed the expression patterns of *B. napus G3BP* (*BnaG3BP*) genes in various tissues/organs in response to *Sclerotinia* disease, blackleg disease, powdery mildew, dehydration, drought, heat, cold, and ABA treatments, and its involvement in seed traits including germination, α-linolenic acid content, oil content, and yellow seed. Several *BnaG3BP* DEGs might be regulated by BnaTT1. The qRT-PCR assay validated the inducibility of two cold-responsive *BnaG3BP* DEGs. This study will enrich the systematic understanding of *Brassica G3BP* family genes and lay a molecular basis for the application of *BnaG3BP* genes in stress tolerance, disease resistance, and quality improvement in rapeseed.

## 1. Introduction

RasGAPase-activating proteins (SH3 structural domain) binding proteins (G3BPs, also known as Rasputins) are a family of highly conserved RNA-binding proteins that developed during eukaryotic evolution [1]. G3BP is an important component of stress granules (SGs); SGs are generated in the cytoplasm in response to stresses, and might function in the regulation of mRNA metabolism in stress [2]. G3BPs contain four distinct structural domains [3]: a nuclear transporter factor 2 (NTF2)-like region, an acidic and proline-rich region, an RNA recognition motif (RRM), and an arginine- and glycine-rich region (RGG). Currently, there has been very few reports on plant G3BPs, unlike in mammals and Drosophila. Like those in mammals, plant G3BPs are also likely to participate in various cellular processes in which they coordinate signal transduction and post-transcriptional regulation, and meanwhile they can affect the formation of SGs.

G3BPs play important roles in the response to environmental stresses [3]. Based on the coexistence of conserved NTF2-like and RRM domains, eight *G3BP* family genes (*AtG3BPs*) were identified in *Arabidopsis thaliana* [1]. A gene expression analysis of *AtG3BPs* in response to different stresses, including abiotic stress, biotic stress, inducers, hormones, and nutrient starvation, revealed that all abiotic stresses induce gene expression, except oxidative stress, which inhibits gene expression [3]. For example, *AtG3BP1*, *AtG3BP2*, *AtG3BP3*, *AtG3BP5*, and *AtG3BP7* are induced by cold stress. The necrotrophic fungus *Botrytis cinerea* induces the expression of *AtG3BP3* and *ATG3BP7*; *Pseudomonas syringae* induces the expression of *AtG3BP3* and represses the expression of *AtG3BP2* [3]. Gibberellic acid (GA) induces the expression of *AtG3BP2* and *AtG3BP3* but suppresses *AtG3BP7* expression, and abscisic acid (ABA) induces the expression of *AtG3BP3* and *AtG3BP5* [3]. Now there is no report on the involvement of G3BPs in seed traits, including seed oil content, seed fatty acid composition, yellow seed, seed germination, and seed development.

Rapeseed (*Brassica napus*, oilseed rape) is one of the most important oilseed crops worldwide, which provides about 13–16% of edible vegetable oil for humans [4]. *Brassica* is one of the most economically valuable genera in the Brassicaceae, with a wide variety of plants and with many subspecies, varieties, and cultivars, which have high agronomic and economic importance [5,6,7]. *A. thaliana* is closely related to *Brassica* species. Currently, only *A. thaliana G3BPs* have been identified at a whole-genome scale and have been reported to play potential roles in response to abiotic/biotic stresses [1,3]. The *G3BP* family genes in other plants including *Brassica* species have no genome-wide or function studies. In this study, the *G3BP* family genes from six U’s triangle *Brassica* species including *B. rapa*, *B. oleracea*, *B. nigra*, *B. napus*, *B. juncea*, and *B. caritana* are systematically identified at the whole-genome level. Based on transcriptome data, the expression patterns of *B. napus G3BP* (*BnaG3BP*) genes in various tissues/organs in response to *Sclerotinia* disease, blackleg disease, powdery mildew, dehydration, drought, heat, cold, and ABA treatments, and their involvement in seed oil content, seed fatty acid composition, and yellow seed traits, were analyzed. This will provide an important molecular basis for further study of the biological function of *G3BPs* in *Brassica* species, and for the application of *BnaG3BP* genes in stress tolerance, disease resistance, and quality improvement in rapeseed.

## 2. Results

### 2.1. Identification of G3BPs in U’s Triangle Brassica Species

A total of 27, 23, and 20 *G3BP* family genes were identified in the allotetraploid *Brassica* species, *B. napus*, *B. juncea*, and *B. caritana*, respectively (Table S1). There existed a total of 13, 13, and 10 *G3BP* family genes in the diploid progenitors, *B. rapa*, *B. oleracea*, and *B. nigra*, respectively. Here, eight *G3BP* family genes were also identified in *A. thaliana*, which is consistent with previously reported findings [1]. The gene sequences of the *G3BP* genes in these seven species ranged from 1564 bp to 8479 bp in length, whereas their coding regions varied from 1188 bp to 2628 bp. In addition, the G3BP proteins in these seven species ranged from 395 aa to 875 aa in length, their theoretical molecular weight varied from 43.11 kDa to 83.68 kDa, and their theoretical *pI* values ranged from 4.74 to 9.74. The subcellular localization prediction showed that the G3BP proteins of these seven species were mainly located in the chloroplasts, cytoplasm, mitochondria, nucleus, and vacuoles.

### 2.2. Phylogenetic Relationship Analysis

**Figure 1:**
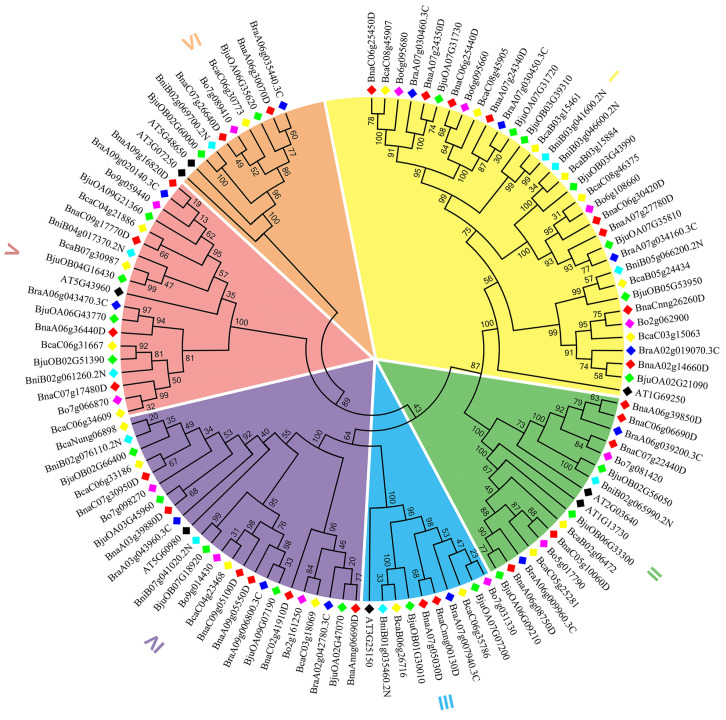
Phylogenetic tree of G3BP proteins among *B. napus*, *B. juncea*, *B. caritana*, *B. rapa*, *B. oleracea*, *B. nigra*, and *A. thaliana*.

Phylogenetic analyses showed that the 114 G3BP family members from seven species were grouped into six clades based on sequence similarity: I (34), II (17), III (10), IV (24), V (19), and VI (10) (Figure 1). The number of each clade in these seven species is shown in detail in Table 1. For the eight *G3BP* genes in *A. thalina*, only the *AT3G07250* ortholog was absent in the six U’s triangle *Brassica* species, which might support the hypothesis that *AT3G07250* gene loss occurred in *Brassica* species after their separation from *A. thalina* in the evolutionary process.

### 2.3. Analysis of Gene Structure and Conserved Motifs

**Figure 2:**
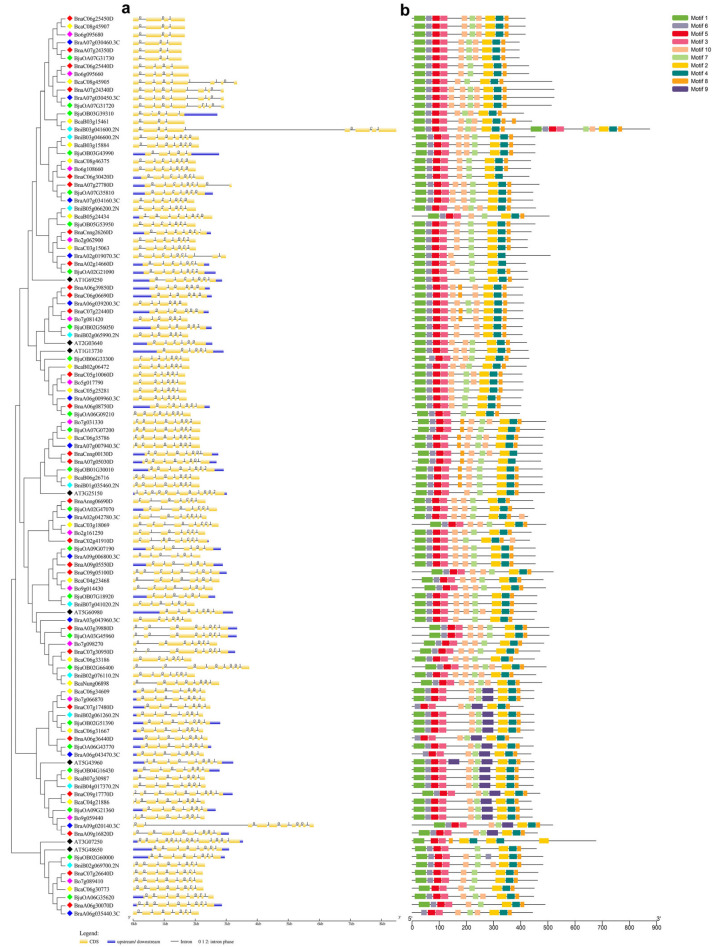
Gene exon–intron structure (**a**) and protein conserved motifs (**b**) of *G3BP* family members in *B. napus*, *B. juncea*, *B. caritana*, *B. rapa*, *B. oleracea*, *B. nigra*, and *A. thaliana*.

The gene structures and protein motifs of the *G3BP* family genes in *Brassica* species and *A. thaliana* were analyzed using GSDS 2.0 and the MEME online database, respectively. The results showed that there existed 3–14 introns in the *G3BP* genes in these seven species (Figure 2). The G3BP proteins from these seven species contained 10 protein conserved motifs (Motifs 1–10), and they were highly conserved in each G3BP clade.

### 2.4. Chromosome Location, Gene Duplication, and Selection Pressure Analysis

In total, 23 *BnaG3BPs* were unevenly located in the 10 chromosomes of *B. napus*, and the remaining 4 *BnaG3BPs* were mapped in unknown sites (Table S1). A total of 23 *BjuG3BPs* were unevenly distributed in the 12 chromosomes of *B. juncea*. There existed 19 *BcaG3BPs* that were unevenly distributed in the 10 chromosomes of *B. carinata* and 1 *BcaG3BP* in an unknown site. In both *B. rapa* and *B. oleracea*, 13 *BraG3BPs* and 13 *BolG3BPs* were unevenly mapped in five chromosomes. In *B. nigra*, 10 *BniG3BPs* were located in six chromosomes. The gene duplication analysis showed that there existed a total of 10 duplicated gene pairs for *G3BPs* in diploid *Brassica* species, which contained eight segmental duplications and two tandem duplications (Table S2). For *G3BPs* in allotetraploid *Brassica* species, a total of 75 duplicated gene pairs were identified, which included 68 segmental duplications and 7 tandem duplications. For these 85 duplicated gene pairs in the *G3BPs* of six *Brassica* species, all Ka/Ks ratios were below 1, suggesting that purifying selection might occur in the evolution process.

### 2.5. Prediction of Upstream miRNAs

The function of miRNAs lies in regulating the post-transcription expression of genes, and thus we predicted the miRNAs targeting *G3BPs* in six *Brassica* species and *A. thaliana* using the psRNATarget online database. As illustrated in Table S3, a total of 96 miRNAs were identified to target 63 *G3BPs* from these seven species, which primarily inhibit mRNA expression through a cleavage effect (96.87%). The *mtr-miR2673a* and *mtr-miR2673b* both were predicted to target 34 *G3BPs* in seven species, whereas the remaining miRNAs were predicted to target 1 to 10 *G3BPs* in seven species.

### 2.6. Analysis of SSR Loci

To analyze the distributions of SSR loci in *G3BP* genes from six *Brassica* species, we identified their SSR motifs using the online MISA-web database. As shown in Table S4, a total of 21 SSR loci were identified in the *G3BP* genes in six *Brassica* species and *A. thaliana*, which included seven types: mono- (p1, 2), di- (p2, 5), tri- (p3, 3), tetra- (p4, 4), penta- (p5, 3), and nonanucleotide (p9, 2), as well as compound type (c, 2). There existed nine SSR loci in seven *BnaG3BPs*, two in two *BjuG3BPs*, four in three *BcaG3BPs*, one in one *BraG3BP*, three in three *BolG3BPs*, and one in one *BniG3BP*.

### 2.7. Expression Patterns of BnaG3BP Genes in Various Tissues/Organs

**Figure 3:**
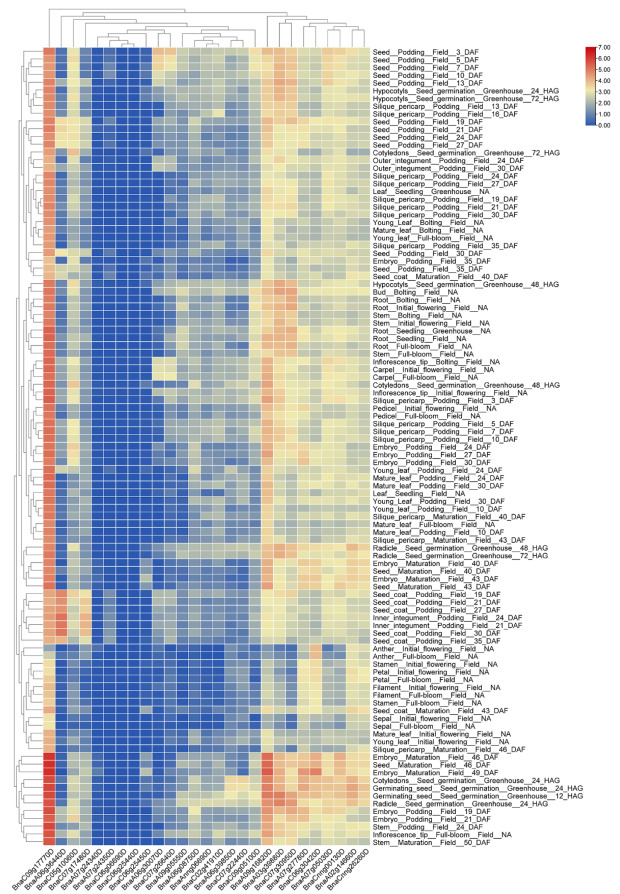
Expression patterns of *BnaG3BP* genes in various tissues/organs of *B. napus*. DAF, days after flowering; HAG, hours after germination.

To examine the organ specificity of *BnaG3BP* expression, based on the BrassicaEDB database, we analyzed the expression patterns of *BnaG3BPs* in 103 tissues and organs of the seven growth and developmental periods of *B. napus*. As shown in Figure 3 and Table S5, there was almost no expression in 103 tissues for *BnaA07g24350D*, *BnaC06g25450D*, *BnaA07g24340D*, *BnaC06g06690D*, and *BnaC06g25440D*. The *BnaA07g24350D* FPKM values were 1–1.91 only in seven tissues, *BnaC06g25450D* FPKM values were 1–3.71 only in eight tissues, *BnaA07g24340D* FPKM values were less than 0.75, and FPKM values of *BnaC06g06690D* and *BnaC06g25440D* were almost less than 0.05. The rest of the *BnaG3BP* genes were expressed in most tissues and organs (FPKM > 1), for example, the *BnaC09g17770D* FPKM values ranged from 2.18 to 77.68 in 103 tissues (Figure 3). *BnaG3BP* genes from clade III and V both had higher expression levels in various organs, while those from clade II and VI both had very lower expression levels. Some of *BnaG3BPs* in Clade I and IV possessed higher expressions and their remaining genes were expressed at lower levels.

### 2.8. Expression Patterns of BnaG3BP Genes in Response to Biotic Stresses

To detect the potential roles of *BnaG3BP* in plant disease defense, we analyzed their expression patterns in response to biotic stresses including *Sclerotium* stem rot (*Sclerotium sclerotiorum*), blackleg disease (*Leptosphaeria maculans*), and powdery mildew (*Erysiphe cruciferarum*), based on RNA-seq data (Tables S6–S8).

**Figure 4:**
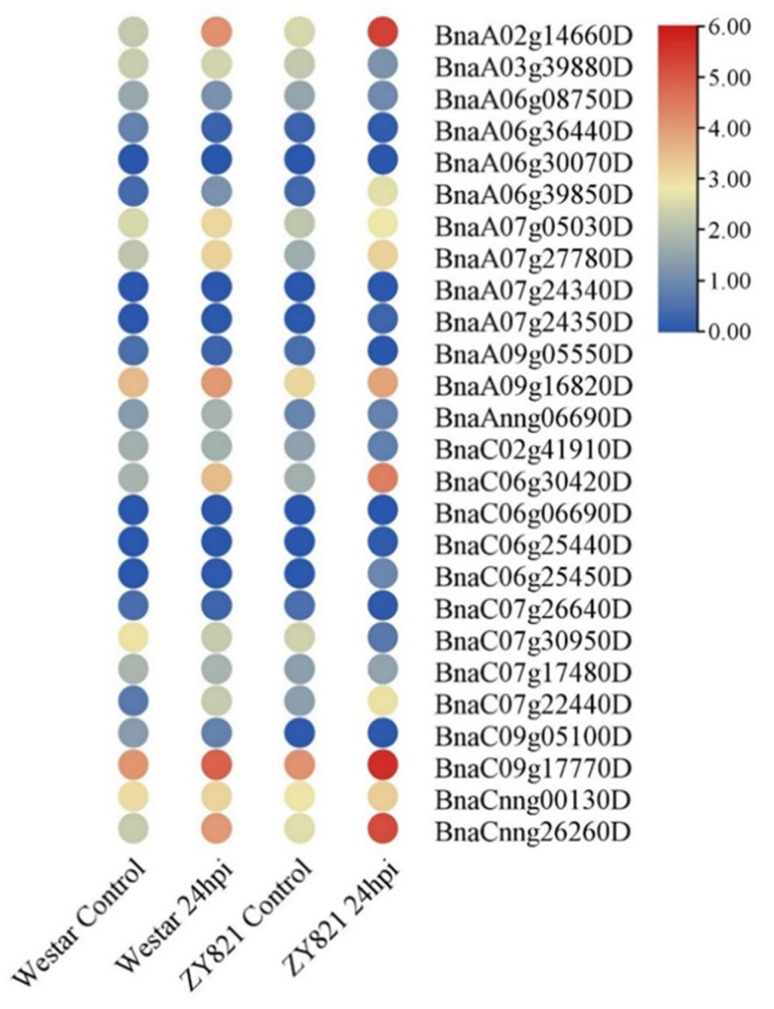
Expression patterns of *BnaG3BP* genes in the leaves of susceptible (Westar) and tolerant (ZY821) genotypes of rapeseed infected with *S. sclerotiorum* at 24 h post-inoculation (24 hpi).

In response to *S. sclerotiorum*, there were six differentially expressed genes (DEGs) of *BnaG3BP* (*BnaA02g14660D*, *BnaA06g39850D*, *BnaA07g27780D*, *BnaC06g30420D*, *BnaC07g22440D*, and *BnaCnng26260D*) in a susceptible host (Westar), all of which were up-regulated (Figure 4). There were nine *BnaG3BP* DEGs in a resistant host (ZY821), of which seven (*BnaA02g14660D*, *BnaA06g39850D*, *BnaA07g27780D*, *BnaC06g30420D*, *BnaC07g22440D*, *BnaC09g17770D*, and *BnaCnng26260D*) were up-regulated, and two (*BnaA03g39880D* and *BnaC07g30950D*) were down-regulated. There were six commonly up-regulated *BnaG3BP* DEGs in both resistant and susceptible hosts. There was no commonly down-regulated DEGs. There existed three *BnaG3BP* DEGs that were only in the resistant host, of which one was up-regulated and two were down-regulated. There was no *BnaG3BP* DEG that was only in the susceptible host.

**Figure 5:**
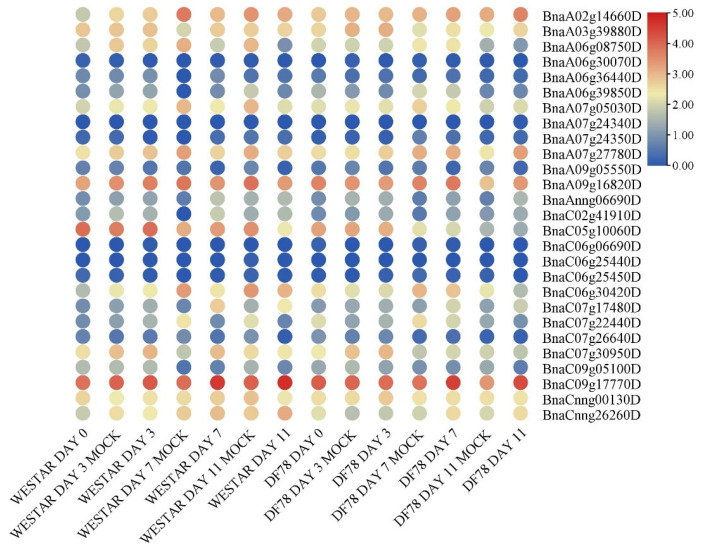
Expression patterns of *BnaG3BP* genes in the cotyledons of rapeseed susceptible (Westar) and resistant (DF78) lines at 0 day, 3 days, 7 days, and 11 days post-*L. maculans* inoculation.

In the response of the susceptible host to *L. maculans*, there were no *BnaG3BP* DEGs 3 days after inoculation, and there were five *BnaG3BP* DEGs 7 days after inoculation, of which three were up-regulated (*BnaAnng06690D*, *BnaC07g17480D*, and *BnaC07g30950D*) and two were down-regulated (*BnaA06g08750D* and *BnaC07g22440D*).There were seven *BnaG3BP* DEGs 11 days after inoculation, of which one was up-regulated (*BnaC07g17480D*) and six were down-regulated (*BnaA07g05030D*, *BnaA06g39850D*, *BnaC05g10060D*, *BnaA06g08750D*, *BnaC07g22440D*, and *BnaC09g05100D*). In the response of the tolerant host to *L. maculans*, there were two *BnaG3BP* DEGs three days after inoculation, of which one was up-regulated (*BnaAnng06690D*), and one was down-regulated (*BnaC07g22440D*). There were three *BnaG3BP* DEGs (*BnaAnng06690D*, *BnaC07g17480D*, and *BnaC02g41910D*) seven days after inoculation, of which all were up-regulated. There were five *BnaG3BP* DEGs 11 days after inoculation, of which four were up-regulated (*BnaAnng06690D*, *BnaC07g17480D*, *BnaA07g27780D*, and *BnaC09g17770D*) and one was down-regulated (*BnaC09g05100D*) (Figure 5).

**Figure 6:**
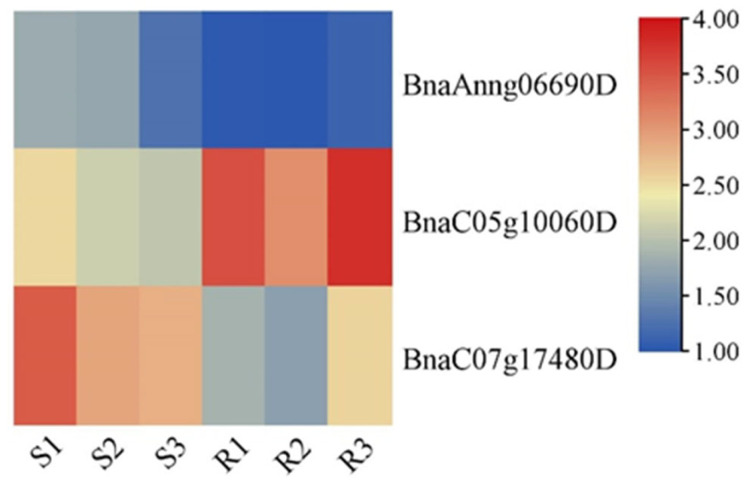
Expression patterns of *BnaG3BP* genes in rapeseed susceptible (S) and resistant (R) lines after powdery mildew inoculation.

In response to *E. cruciferarum*, the FPKM values of a total of three *BnaG3BPs* were obtained from the transcriptome data. After inoculation with powdery mildew, R (resistant) vs. S (susceptible) had two *BnaG3BP* DEGs, of which *BnaC05g10060D* was up-regulated and *BnaC07g17480D* was down-regulated (Figure 6).

### 2.9. Expression Patterns of BnaG3BP Genes in Response to Abiotic Stresses

**Figure 7:**
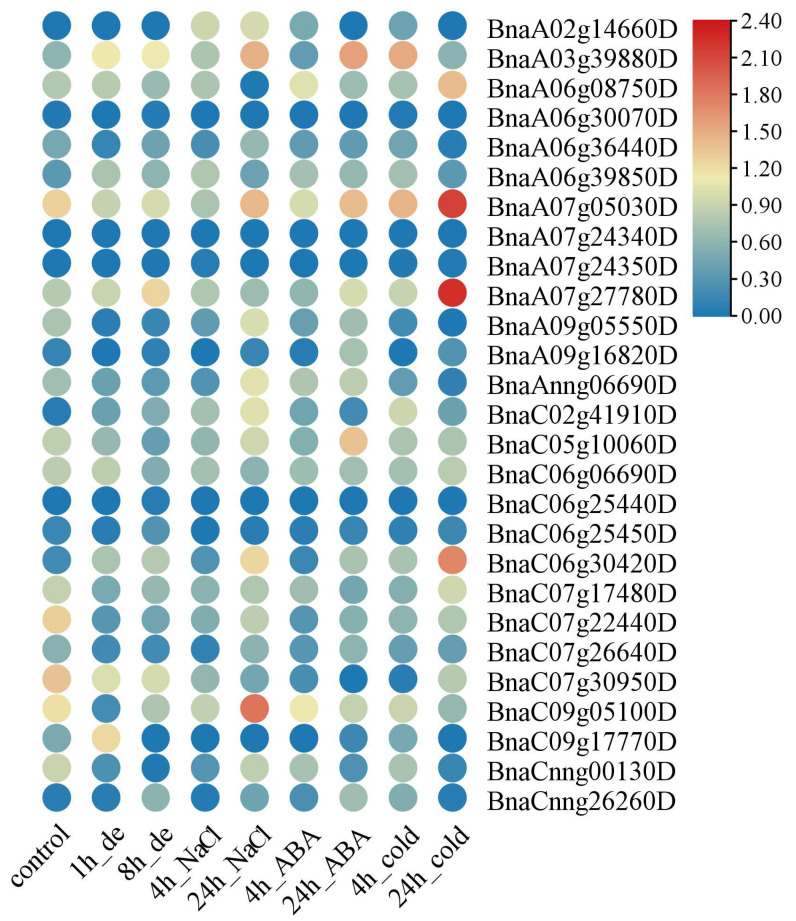
Expression patterns of *BnaG3BP* genes in 3-week-old rapeseed plants under multiple abiotic stresses. Dehydration (1 h and 8 h), ABA (25 μM; 4 h and 24 h), NaCl (200 mM; 4 h and 24 h), and cold (4 °C, 4 h and 24 h).

**Figure 8:**
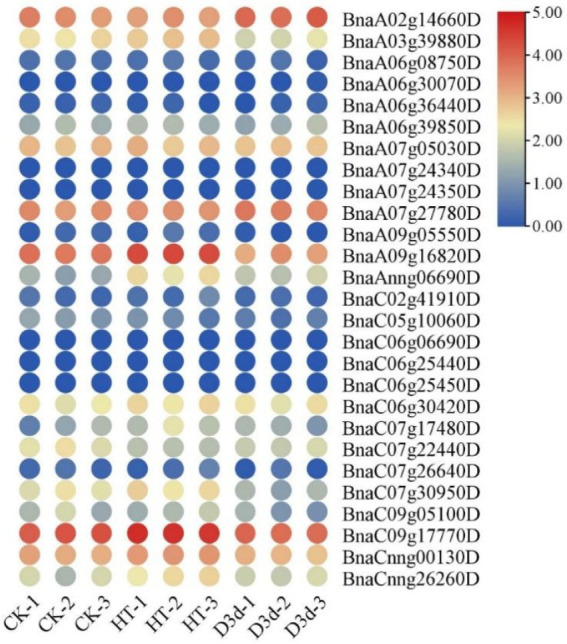
Expression patterns of *BnaG3BP* genes under heat and drought conditions. D3d, drought (withdrawing water) treated for 3 days. HT, heat treated at temperature of 40 °C for 3 h.

To examine the roles of *BnaG3BP* genes in response to abiotic stresses, we analyzed their expression patterns under cold, salt, dehydration, heat, drought, and ABA treatments based on RNA-seq data (Tables S9 and S10). Under dehydration stress, there were four *BnaG3BP* DEGs at 1 h after treatment, of which two were up-regulated (*BnaC09g17770D* and *BnaA03g39880D*) and two were down-regulated (*BnaC07g22440D* and *BnaC09g05100D*); there were two *BnaG3BP* DEGs for 8 h of treatment, in which one was up-regulated (*BnaA03g39880D*) and one was down-regulated (*BnaC07g22440D*) (Figure 7). Under NaCl stress, there were three *BnaG3BP* DEGs (*BnaA07g05030D*, *BnaC07g22440D*, and *BnaC07g30950D*), in which all were down-regulated at 4 h of treatment; there were four *BnaG3BP* DEGs (*BnaC06g30420D*, *BnaC02g41910D*, *BnaA03g39880D*, and *BnaC09g05100D*), of which all were up-regulated. Under ABA treatment, there existed two *BnaG3BP* DEGs (*BnaC07g22440D* and *BnaC07g30950D*) at 4 h, of which all were down-regulated; there were two *BnaG3BP* DEGs at 24 h, with one being up-regulated (*BnaA03g39880D*) and the other down-regulated (*BnaC07g22440D*). Under cold stress, there were three *BnaG3BP* DEGs, in which one gene was up-regulated (*BnaA03g39880D*) and two were down-regulated (*BnaC07g22440D* and *BnaC07g30950D*) after 4 h of treatment. There were seven *BnaG3BP* DEGs at 24 h of cold treatment, of which four were up-regulated (*BnaC06g30420D*, *BnaA07g27780D*, *BnaA07g05030D*, and *BnaA06g08750D*) and three were down-regulated (*BnaC07g22440D*, *BnaC07g30950D*, and *BnaC09g05100D*). Under heat stress, there were two *BnaG3BP* DEGs (*BnaAnng06690D* and *BnaC07g17480D*), which were both up-regulated (Figure 8). Under drought stress, there existed two *BnaG3BP* DEGs (*BnaC07g30950D* and *BnaC05g10060D*), which were both down-regulated.

### 2.10. Expression Patterns of BnaG3BP Genes Involved in Seed Germination Traits

**Figure 9:**
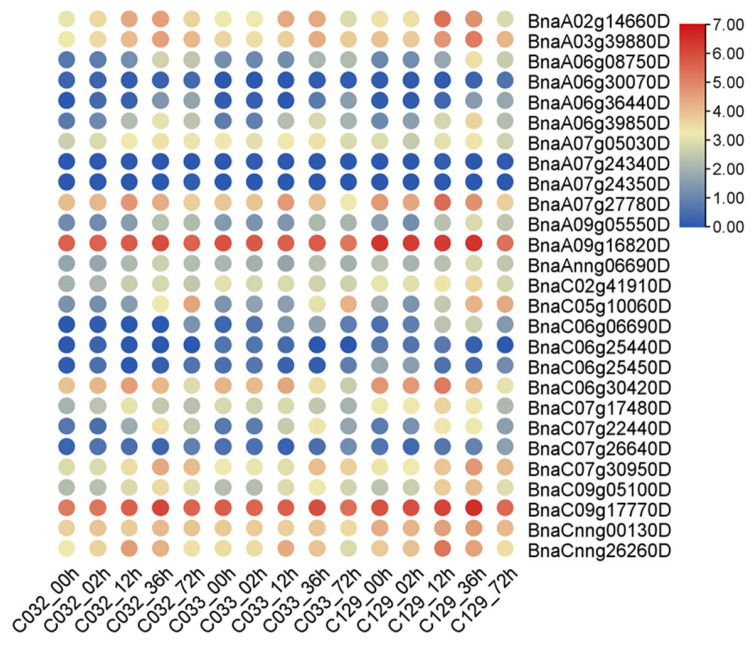
Expression patterns of *BnaG3BP* genes in seed germination process of three selected accessions (low, medium or high germination rates; C032, C033 and C129, respectively) at 0, 12, 24, 48 and 72 h after imbibition.

To investigate the potential roles of *BnaG3BPs* in regulating seed germination traits, we analyzed their expression patterns during the seed germination process of three selected accessions (low, medium, or high germination rates; C032, C033, and C129, respectively) at 0, 12, 24, 48, and 72 h after imbibition, based on RNA-seq data (Table S11 and Figure 9). In total, there existed 14, 11, and 17 *BnaG3BP* DEGs in the seed germination process of C032, C033, and C129, respectively, in which there were 3 common *BnaG3BP* DEGs. In the C032, C033, and C129 accessions, there existed one, two, and four down-regulated *BnaG3BP* DEGs, respectively. There were 13, 9, and 13 up-regulated *BnaG3BP* DEGs in the C032, C033, and C129 accessions, respectively. This result shows that these *BnaG3BP* DEGs might be involved in the regulation of seed germination traits.

### 2.11. Expression Patterns of BnaG3BP Genes Involved in Yellow Seed Trait

**Figure 10:**
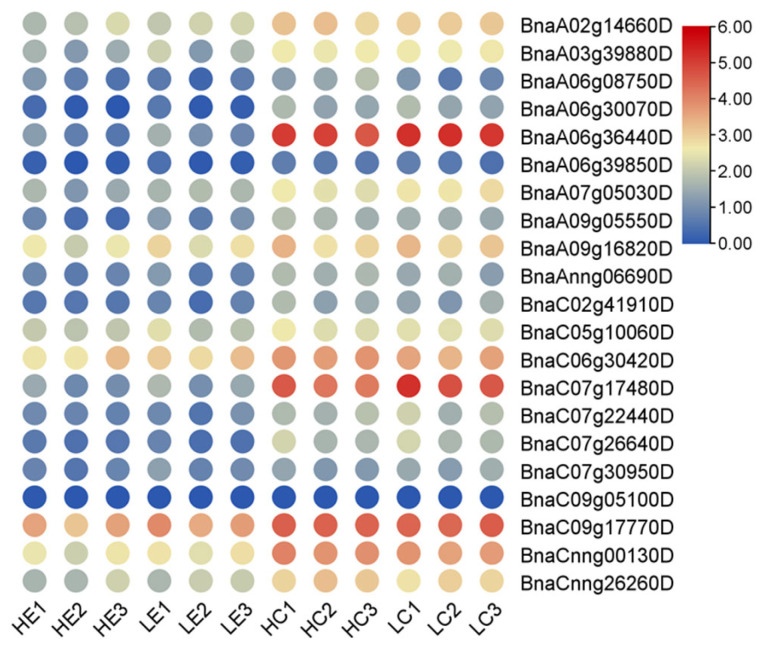
Expression patterns of *BnaG3BP* genes involved in yellow seed traits. Yellow seed coats at LC1 (35 days after flowering (DAF)), LC2 (40 DAF), and LC3 (46 DAF); yellow seed embryos at LE1 (35 DAF), LE2 (40 DAF), and LE3 (46 DAF); black seed coats at HC1 (35 DAF), HC2 (40 DAF), and HC3 (46 DAF); and black seed embryos a HE1 (35 DAF), HE2 (40 DAF), and HE3 (46 DAF).

To examine the potential roles of *BnaG3BPs* in yellow seed traits, we analyzed their expression patterns in coats and embryos from black seeds and yellow seeds at 35, 40, and 46 DAF in rapeseed (Table S12 and Figure 10). In total, there were two *BnaG3BP* DEGs (*BnaA06g08750D* and *BnaA02g14660D*, both down-regulated) in the yellow coats vs. the black coats, whereas three *BnaG3BP* DEGs were observed in the yellow embryos vs. the black embryos, of which one was down-regulated (*BnaA06g08750D*) and two were up-regulated (*BnaA07g05030D* and *BnaA09g05550D*). This result suggests that these *BnaG3BP* DEGs might play potential roles in the regulation of yellow seed traits.

### 2.12. Expression Patterns of BnaG3BP Genes Involved in Seed ALA Trait

**Figure 11:**
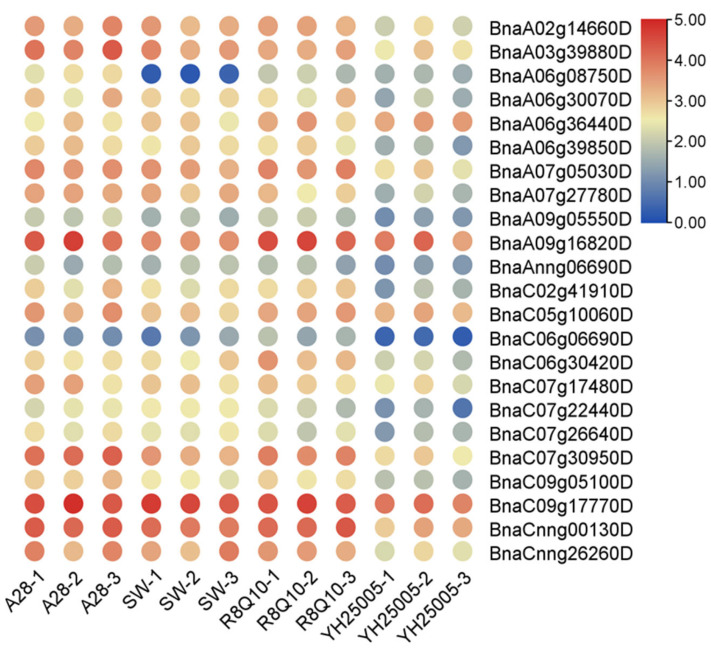
Expression patterns of *BnaG3BP* genes in 24-day-old seed embryos from two rapeseed inbred lines with high (YH25005 and R8Q10)/low (SW and A28) α-linolenic acid traits.

To investigate the potential function of *BnaG3BPs* in regulating seed linolenic acid traits, we analyzed their expression patterns in 24-day-old seed embryos from two rapeseed inbred lines with high (YH25005 and R8Q10) and low (SW and A28) α-linolenic acid (ALA) contents (Table S13). We found that there were 0, 16, 1, and 10 *BnaG3BP* DEGs in R8Q10 vs. A28, YH25005 vs. A28, R8Q10 vs. SW, and YH5005 vs. SW, respectively (Figure 11), in which only the common *BnaG3BP* DEG (*BnaA06g08750D*) in R8Q10 vs. SW and YH5005 vs. SW was up-regulated, and the rest of the *BnaG3BP* DEGs all were down-regulated. This suggests that the *BnG3BP* DEGs are likely to mainly be negative regulators of seed ALA content.

### 2.13. Expression Patterns of BnaG3BP Genes Involved in Seed Oil Content Traits

**Figure 12:**
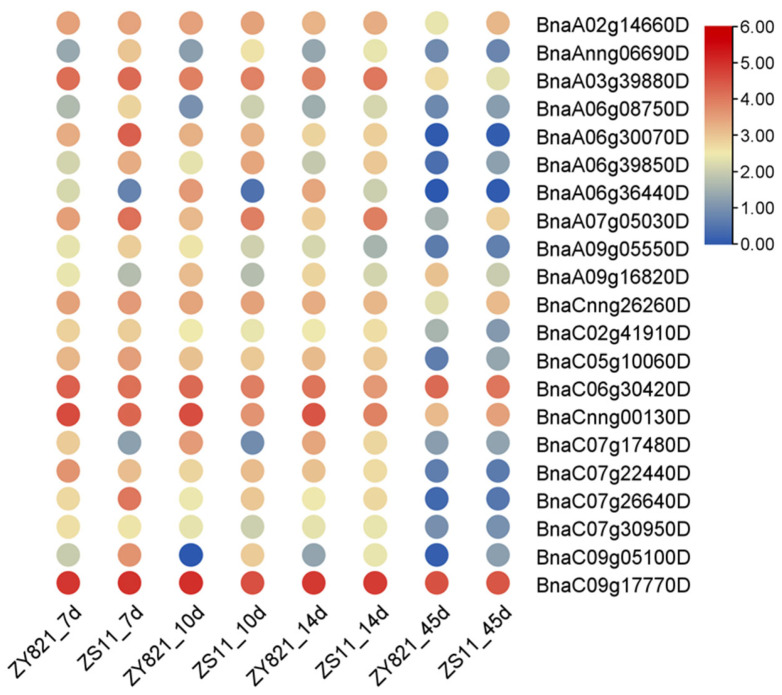
Expression patterns of *BnaG3BP* genes in 7-, 10-, 14- and 45-day-old seeds from two rapeseed cultivars with high (ZS11)/low (ZY821) seed oil content.

To determine the potential roles of *BnaG3BPs* in regulating seed oil content, we analyzed their expression patterns in 7-, 10-, 14- and 45-day-old seeds from two rapeseed cultivars with high (ZS11)/low (ZY821) seed oil contents (Table S14 and Figure 12). In total, there were eight *BnaG3BP* DEGs (two down-regulated and six up-regulated) in ZS11_7d vs. ZY821_7d. There existed six *BnaG3BP* DEGs (three down-regulated and three up-regulated) in ZS11_10d vs. ZY821_10d. There were five *BnaG3BP* DEGs (one down-regulated and four up-regulated) in ZS11_14d vs. ZY821_14d. There existed six *BnaG3BP* DEGs (one down-regulated and five up-regulated) in ZS11_45d vs. ZY821_45d. Several genes kept the same differential expression trends along the seed development process, e.g., *BnaC09g05100D*, *BnaC07g26640D*, *BnaA07g05030D*, *BnaA06g39850D*, *BnaA06g08750D* and *BnaAnng06690D* were successively higher in high-oil ZS11 than in low-oil ZY821, while *BnaA06g36440D*, *BnaA09g16820D* and *BnaC07g17480D* were successively lower in ZS11 than in ZY821.This result shows that these *BnaG3BP* DEGs might be involved in the regulation of seed oil content.

### 2.14. Expression Patterns of BnaG3BP Genes in BnaTT1 Transgenic Lines

TT1s negatively regulate seed oil accumulation but positively regulate flavonoid-proanthocyanidin biosynthesis [8]. To detect whether *BnaG3BPs* are target genes of *B. napus* TT1 (BnaTT1), we analyzed *BnaG3BP* DEGs in the transcriptome data of the *BnaTT1* transgenic lines in our group (unpublished). In total, there existed one and three *BnaG3BP* DEGs in the mid-stage seeds of transgenic rapeseeds over-expressing *pNapA*::*BnaTT1* (NOE) and *pBAN*::*BnaTT1* (BOE), respectively. As shown in Table 2 and Table 3 (reference source data unpublished), after the overexpression of NOE and BOE, these four *BnaG3BP* DEGs (*BnaC05g10060D*, *BnaA03g39880D*, *BnaC07g17480D*, and *BnaC07g30950D*) all were down-regulated, and *BnaC07g17480D* DEG was specifically expressed in the mid-stage seed coat, especially in the inner integument (Figure S1). This suggests that they might be downstream target genes of BnaTT1 and might be involved in the regulation of seed development and metabolism.

### 2.15. Validation of Selected Cold-Responsive Genes

**Figure 13:**
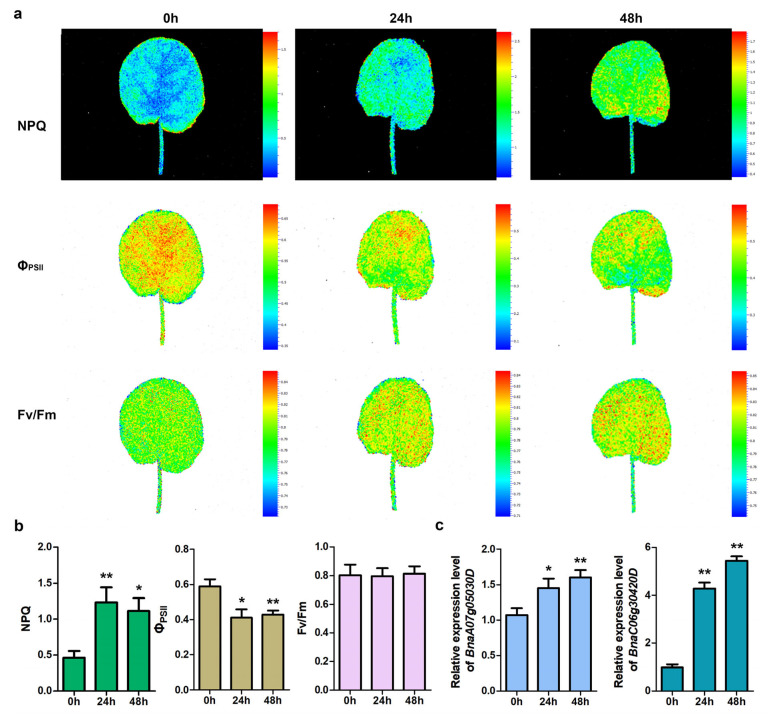
Impact of cold stress on photosynthesis of rapeseed leaves (**a**,**b**), and qRT-PCR expression levels of selected cold-responsive *BnaG3BP* DEGs with *25sRNA* as internal control (**c**). Standard images of Fv/Fm, Φ_PSII_, and NPQ from cold-treated rapeseed leaves at 0, 24, and 48 h. False color scale is used for each parameter. Values represent average ± SD of three biological replicates. Fv/Fm, maximum quantum yield of PSII; Φ_PSII_, effective quantum yield of PSII; and NPQ, non-photochemical quenching. * *p* < 0.05 and ** *p* < 0.01 compared with samples at 0 h.

To validate the accuracy of the transcriptome data, a qRT-PCR analysis was conducted on two representative cold-responsive *BnaG3BP* DEGs. The results showed that these two DEGs were both significantly up-regulated under cold treatment (Figure 13), which is consistent with their RNA-seq results. Additionally, the chlorophyll fluorescence parameters of rapeseed leaves after cold treatment also changed significantly. The NPQ values were up-regulated and the Φ_PSII_ values were down-regulated, suggesting their potential application in cold stress detection at an early-stage.

## 3. Discussion

Substantial studies have showed that RNA-binding proteins (RBPs) play important roles in regulating post-transcriptional changes, which is of great significance for plant growth and development, and defenses against biotic/abiotic stresses [1,9,10,11]. G3BPs belong to a highly conserved family of RBPs in eukaryotic organisms, which function in signal transduction and post-transcriptional gene regulation, and play important roles in the formation of SGs [1,2,3]. Unlike mammals and *Drosophila*, there are limited reports on the function plant G3BPs as well as their roles in the production of SGs [3]. In the TAIR10 reference genome of *A. thaliana*, it had been reported that there exists eight *G3BP* family members [1]. Here, we further validated that there are also the same number of *AtG3BPs* in the TAIR11 genome. According to the U’s triangle model of *Brassica* species, based on natural hybridization, the diploid ancestors *B. rapa* (AA) and *B. oleracea* (CC) putatively produce the allotetraploid *B. napus* (AACC), diploid parents *B. rapa* (AA) and *B. nigra* (BB) putatively generate the allotetraploid *B. juncea* (AABB), and diploid progenitors *B. nigra* (BB) and *B. oleracea* (CC) putatively give rise to *B. caritana* (BBCC) [12,13,14]. In this study, the allotetraploids *B. napus*, *B. juncea*, and *B. caritana* contained 27, 23, and 20 *G3BP* genes, respectively, and a total of 13, 13, and 10 *G3BP* genes were identified in the diploid progenitors *B. rapa*, *B. oleracea*, and *B. nigra*, respectively. Obviously, the total number of members of the *G3BP* family in the three diploid progenitors and three allotetraploid *Brassica* species basically conform to U’s triangle model. However, the total numbers of the *G3BP* family genes from these six *Brassica* species of U’s triangle still need to be further determined when their gap-free T2T pan-genomes all are published.

In *A. thaliana*, the expression profiles of the *AtG3BP* genes were affected by bacterial, fungal, and different abiotic stresses and hormones, suggesting that *AtG3BP*s have potential roles in various stress signaling pathways [3]. In *B. napus*, we also identified a variety of *BnaG3BP* DEGs in responses to *Sclerotinia* disease, blackleg disease, and powdery mildew, suggesting they might be involved in disease resistance by post-transcriptional gene regulation. Moreover, *BnaG3BP* DEGs were identified after treatments of various abiotic stresses including heat, drought, dehydration, NaCl, cold, and ABA, suggesting these *BnaG3BP* DEGs might play potentially important roles in response to abiotic stresses. These results will provide insights for the potential application of *BnaG3BP* genes in the genetic improvement of disease resistance and abiotic stress tolerance in *B. napus*.

Currently, there has been no report on the involvement of *G3BPs* in improvements of seed traits including seed germination, seed oil content, seed ALA content, and yellow seed traits. As for RBPs, there were several literatures regarding the regulation of traits including seed germination, seed development, and seed size. For example, in rice and wheat, *EOG1*, which encodes an RNA-binding protein with two RRMs and one SPOC domain, negatively regulates seed size and weight [15]. In *A. thaliana*, the plant-specific *SCL30a* gene encodes an RNA-binding protein belonging to splicing regulators and it was found that SCL30a affected ABA-related seed traits and retarded germination [16]. In this study, we found that there exists a large number of *BnaG3BP* DEGs that are involved in seed germination, seed oil content, seed ALA content, and yellow seed traits. G3BPs belong to one type of RNA-binding proteins, and thus encoding proteins of these identified *BnaG3BP* DEGs that may impact seed traits might function via post-transcriptional mechanisms such as pre-mRNA processing, alternative splicing, mRNA stabilization, RNA editing, and mRNA transport [11].

TT1 negatively regulates oil accumulation but positively regulates flavonoid-proanthocyanidin biosynthesis [8]. In this study, there existed many *BnaG3BP* DEGs in the pairwise transcriptome comparisons of BOE vs. WT and NOE vs. WT for the mid-stage seeds of *BnaTT1* overexpression plants, suggesting that these *BnaG3BP* DEGs might be target genes for the BnaTT1 transcription factor, and might be involved in seed development and seed metabolism.

## 4. Materials and Methods

### 4.1. Acquisition of the Reference Genomes of Brassica Species and A. thaliana

The reference genomes of *B. napus* (Brana_Dar_V5), *B. rapa* (Brara_Chiifu_V3.0), *B. juncea* (Braju_AU213_V1.0), and *B. nigra* (Brani_Ni100_V2) were downloaded from BRAD database (http://brassicadb.cn/, accessed on 1 March 2023). The reference genomes of *B. oleracea* (BOL), *B. caritana*, and *A. thaliana* (v11) were downloaded from EnsemblPlants database (http://plants.ensembl.org/index.html, accessed on 23 February 2023), *Brassica* genomics database (http://bio2db.com, accessed on 26 March 2023), and TAIR database (http://www.arabidopsis.org/, accessed on 23 February 2023), respectively.

### 4.2. Identification of G3BP Gene Family in Brassica Species and A. thaliana

Since G3BP proteins have NTF2 (PF02136) and RRM_1 (PF00076) conserved domains, hmmsearch was performed to retrieve candidate *G3BP* family genes from seven species, including *Brassica* species and *A. thaliana*, by using the HMMER 3.0 software with NTF2 and NTF2 domains as the bait, respectively. Subsequently, proteins with both PF02136 and PF00076 conserved domains were retained by manual checking as well as validation against the SMART database (http://smart.embl-heidelberg.de/, accessed on 31 March 2023); otherwise, they were considered as non-G3BP proteins, were deleted, and were not further analyzed.

### 4.3. Bioinformatics Analysis

Based on gene sequences and coding region sequences of *G3BPs* from *Brassica* species and *A. thaliana*, their exon–intron structural features were analyzed using the online website GSDS2.0 (http://gsds.cbi.pku.edu.cn/, accessed on 16 April 2023). The conserved motifs in their G3BP proteins were identified using the MEME suite 5.5.2 database (http://meme-suite.org/tools/meme, accessed on 16 April 2023) with default parameters. The theoretical isoelectric point (pI) and molecular weight (Mw) of their G3BP proteins were calculated using the ProtParam website (https://web.expasy.org/protparam/, accessed on 17 April 2023). Using the WoLF PSORT II database (https://www.genscript.com/tools/wolf-psort, accessed on 18 April 2023) and the Plant-mPLoc database (http://www.csbio.sjtu.edu.cn/bioinf/plant-multi/, accessed on 18 April 2023), subcellular localizations of their G3BP proteins were predicted. SSR motifs in their *G3BP* genes were predicted on MISA-web database (https://webblast.ipk-gatersleben.de/misa/, accessed on 20 March 2025). The upstream miRNA targeting their *G3BP* genes were predicted on psRNATarget according to the default parameters with the expected value of 3 (https://www.zhaolab.org/psRNATarget/, accessed on 20 March 2025).

### 4.4. Phylogenetic Analysis

The sequences of G3BP family proteins in seven species were multi-aligned using MAFFT7 software, and then neighbor-joining (NJ) phylogenetic tree was constructed using MEGA7 software in *p*-distance mode and their reliability was tested using bootstrap analysis (1000 replicates).

### 4.5. Expression Profiling Based on Transcriptome Data

The transcriptome data of 103 tissues and organs of rapeseed at seven growth and development stages were obtained from BrassicaEDB database [17] (https://brassica.biodb.org/, accessed on 15 April 2023). The NCBI database (https://www.ncbi.nlm.nih.gov/, accessed on 9 April 2023) provided transcriptome data of rapeseed in response to *Sclerotinia* stem pot (GSE81545) [18], blackleg (GSE77723) [19], and powdery mildew (GSE188377) [20] diseases, as well as heat and drought stresses (GSE156029), seed germination trait (GSE137230) [21], seed ALA content trait (GSE186952) [22], and yellow-seed trait (PRJNA597958) [23]. From the NGDC database (https://ngdc.cncb.ac.cn/, accessed on 14 September 2024), transcriptome data of rapeseed in response to dehydration, cold, salt, and ABA treatments (CRA001775) [24], and involvement in seed oil content (PRJCA001246) [25] were retrieved. *BnaG3BP* was considered as differentially expressed gene (DEG) if |FPKM/TPM| ≥ 1 and |log2FoldChange| ≥ 1. TBtools/TBtools-II [26] was used to perform cluster analysis and heatmap creation. Our group have obtained RNA-seq data for mid-stage seeds of transgenic rapeseed overexpressing *BnaTT1* and the transcriptome data were mined to identify *BnaG3BP* DEGs.

### 4.6. The qRT-PCR Analysis

Total RNAs were extracted from the seedling leaves of rapeseed Westar cultivar at 0, 24, and 48 h under cold stress (4◦C), using the RNAsimple Total RNA Kit (DP419, Tiangen, Beijing, China). First-strand total cDNA was produced using the PrimeScript Reagent Kit with gDNA Eraser (RR047A, Takara, Dalian, China). The qRT-PCR assay was performed using TB Green Premix Ex Taq II (Tli RNaseH Plus) (RR820A, Takara, Dalian, China) on a CFX Real-time PCR System (Bio-Rad, Irvine, CA, USA). Each reaction was performed with three replicates. Two representative cold-responsive *BnaG3BP* DEGs, *BnaA07g05030D* and *BnaC06g30420D*, were chosen to perform the qRT-PCR assay with *25sRNA* as internal control (Table S15) as previously described by our group [27].

### 4.7. Chlorophyll Fluorescence Imaging

The fluorescence parameters and chlorophyll fluorescence images of the rapeseed seedling leaves at 0, 24, and 48 h under cold stress were analyzed on the plant phenotype imager (a chlorophyll fluorescence imaging system FluorCam7.0; Photon Systems Instruments, Brno, Czech Republic; device no. 20A00005).

## 5. Conclusions

In this study, a total of 114 *G3BP* genes were identified in six *Brassica* species of U’s triangle (*B. napus*, *B. juncea*, *B. caritana*, *B. rapa*, *B. oleracea*, and *B. nigra*) and *A. thaliana*. Their gene structure, protein motifs, gene duplication type, phylogeny, subcellular localization, SSR loci, and upstream miRNAs were then analyzed. Based on transcriptome data, we also analyzed the expression patterns of *BnaG3BP* genes in various tissues/organs and in response to *Sclerotinia* disease, blackleg disease, powdery mildew, dehydration, drought, heat, cold, and ABA treatments. The RNA-seq data showed that differential expression genes of *BnaG3BP* existed after the overexpression of *BnaTT1* in rapeseed. The qRT-PCR assay validated the inducibility of two representative cold-responsive *BnaGBP* DEGs. This study will promote the systematic understanding of the *G3BP* family genes of U’s triangle *Brassica* species and lay a molecular foundation for the potential application of *BnaG3BP* DEGs in stress tolerance, disease resistance, and seed quality traits in rapeseed.

## Figures and Tables

**Table 1:** The total number of *G3BP* genes in each clade of *B. napus*, *B. juncea*, *B. caritana*, *B. rapa*, *B. oleracea*, *B. nigra*, and *A. thaliana*.

Category	*B. napus*	*B. juncea*	*B. caritana*	*B. rapa*	*B. oleracea*	*B. nigra*	*A. thaliana*
Total	27	23	20	13	13	10	8
I	8	7	7	4	4	3	1
II	5	3	2	2	2	1	2
III	2	2	2	1	1	1	1
IV	6	5	4	3	3	2	1
V	4	4	4	2	2	2	1
VI	2	2	1	1	1	1	2

**Table 2:** *BnaG3BP* DEG in transgenic rapeseeds (Westar cultivar) overexpressing *pNapA*::*BnaTT1* (NOE, 20 days after pollination (DAP) seeds, seed embryo-specific promoter).

Gene ID	NOE_Count	WT_Count	log2FC	*p* Value	*p* Adj
*BnaC05g10060D*	67.51	207.70	−1.62	4.25 × 10^−8^	5.58 × 10^−7^

**Table 3:** *BnaG3BP* DEGs in transgenic rapeseeds (Westar cultivar) overexpressing *pBAN*::*BnaTT1* (BOE, 20 DAP seeds, seed coat-specific promoter).

Gene ID	BOE_Count	WT_Count	log2FC	*p* Value	*p* Adj
*BnaA03g39880D*	100.39	205.50	−1.03	0.00	0.01
*BnaC07g17480D*	76.33	172.32	−1.17	0.00	0.01
*BnaC07g30950D*	92.37	190.97	−1.05	0.00	0.01

## Data Availability

All additional datasets supporting the findings of this study are included within the article and Supplementary Materials.

## Supplementary Materials

The following supporting information can be downloaded at https://www.mdpi.com/article/10.3390/plants14142247/s1. Table S1. Basic information of *G3BP* family genes in six *Brassica* species and *A. thaliana*; Table S2. Gene duplication types and *Ka*/*Ks* analysis for duplicated gene pairs of *G3BP* family genes in six *Brassica* species; Table S3. Basic information of predicted miRNAs targeting *G3BPs* in six *Brassica* species and *A. thaliana*; Table S4. Basic information of predicted SSR loci in *G3BP* family genes from six *Brassica* species and *A. thaliana*; Table S5. The FPKM values of *BnaG3BP* genes in various tissues/organs; Table S6. The FPKM values of *BnaG3BP* genes in response to *Sclerotium sclerotiorum*; Table S7. The FPKM values of *BnaG3BP* genes in response to *Leptosphaeria maculans*; Table S8. The FPKM values of *BnaG3BP* genes in response to *Erysiphe cruciferarum*; Table S9. The average FPKM values of *BnaG3BP* genes in response to multiple abiotic stresses; Table S10. The FPKM values of *BnaG3BP* genes in response to heat and drought; Table S11. The TPM values of *BnaG3BP* genes in seed germination processes; Table S12. The TPM values of *BnaG3BP* genes involved in the yellow seed trait; Table S13. The FPKM values of *BnaG3BP* genes involved in the seed ALA content trait; Table S14. The TPM values of *BnaG3BP* genes involved in seed oil content traits; Table S15. The qRT-PCR primers used in this study; Figure S1. The eFP heatmap (showing FPKM values) of *BnaG3BP* DEGs that might be regulated by BnaTT1 on BrassicaEDB database.
