# Supplementary material for: Genome-Wide Identification of G3BP Family in U’s Triangle Brassica Species and Analysis of Its Expression in B. napus
Source: Plants (Basel). 2025 Jul 21;14(14):2247. doi: 10.3390/plants14142247 (PMC12299536; doi:10.3390/plants14142247)
Supplement: Supplementary file 1 [file plants-14-02247-s001.zip › Supplementary Materials-Figure S1. The eFP heatmap (showing FPKM values) of BnaG3BP DEGs that might be regulated by BnaTT1 on BrassicaEDB database.pdf]

Figure S1 The eFP heatmap (showing FPKM values) of BnaG3BP DEGs that might be regulated by BnaTT1 on BrassicaEDB database

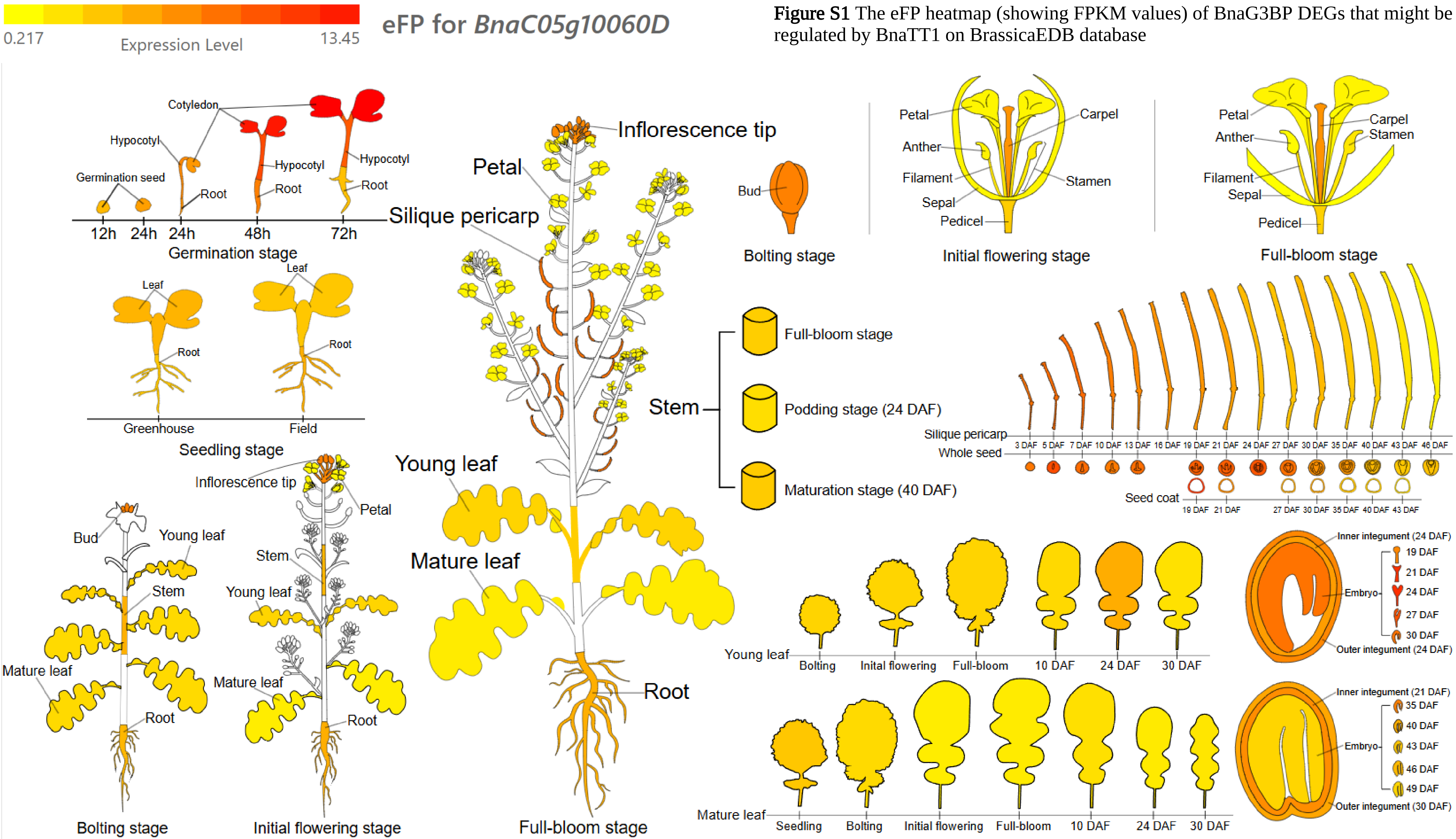

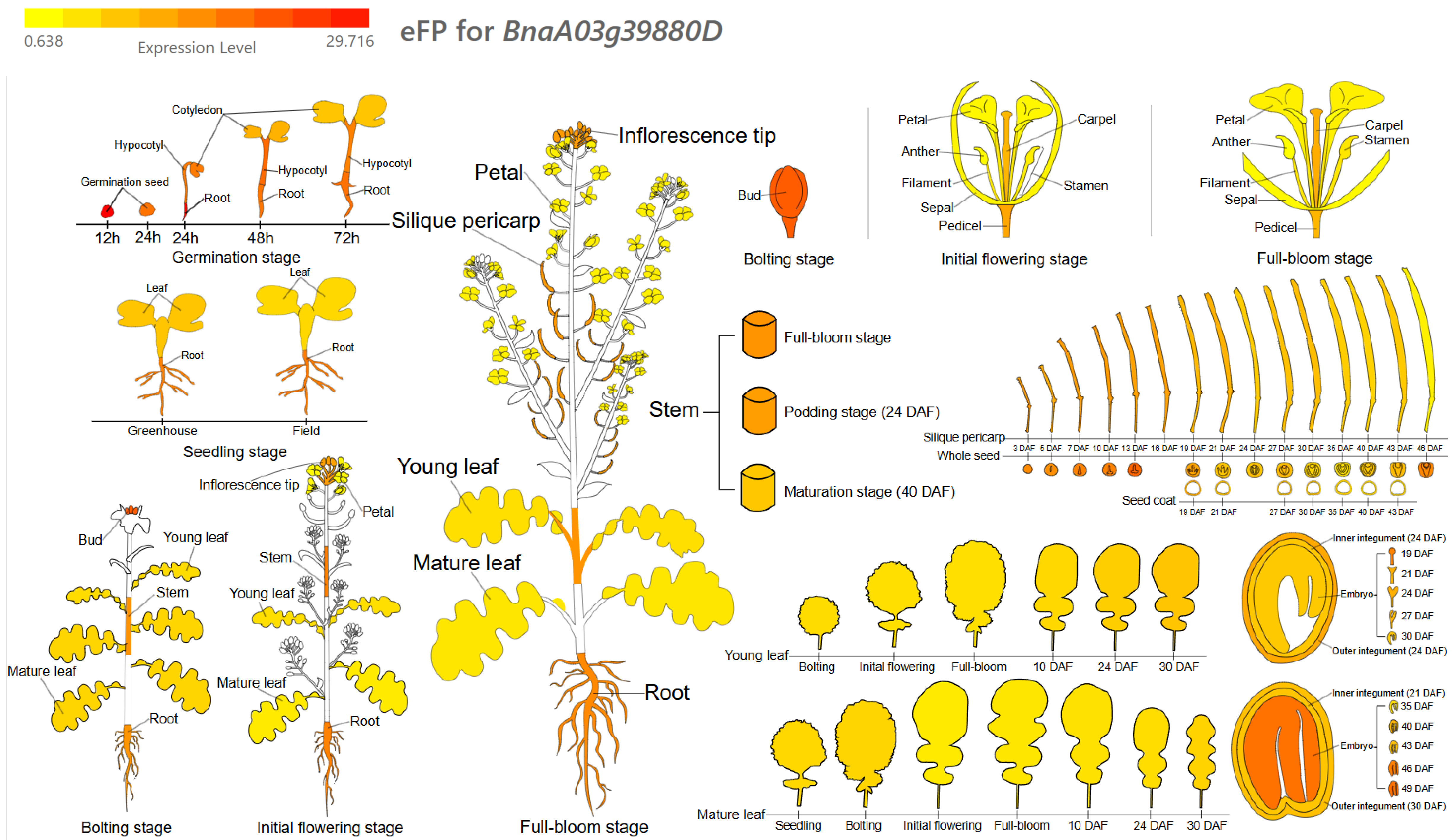

0.638
Expression Level
29.716
eFP for BnaA03g39880D
Cotyledon
Hypocotyl
Germination seed
Root
Hypocotyl
Root
Hypocotyl
Root
12h
24h
24h
48h
72h
Germination stage
Leaf
Root
Leaf
Root
Greenhouse
Field
Seedling stage
Inflorescence tip
Petal
Silique pericarp
Young leaf
Mature leaf
Stem
Root
Full-bloom stage
Bud
Young leaf
Stem
Mature leaf
Root
Bolting stage
Inflorescence tip
Petal
Stem
Young leaf
Mature leaf
Root
Initial flowering stage
Bud
Bolting stage
Petal
Anther
Filament
Sepal
Pedicel
Carpel
Stamen
Initial flowering stage
Petal
Anther
Filament
Sepal
Pedicel
Carpel
Stamen
Full-bloom stage
Full-bloom stage
Podding stage (24 DAF)
Maturation stage (40 DAF)
Silique pericarp
Whole seed
3 DAF
5 DAF
7 DAF
10 DAF
13 DAF
16 DAF
19 DAF
21 DAF
24 DAF
27 DAF
30 DAF
35 DAF
40 DAF
43 DAF
46 DAF
Seed coat
19 DAF
21 DAF
27 DAF
30 DAF
35 DAF
40 DAF
43 DAF
Young leaf
Bolting
Inital flowering
Full-bloom
10 DAF
24 DAF
30 DAF
Mature leaf
Seedling
Bolting
Initial flowering
Full-bloom
10 DAF
24 DAF
30 DAF
Inner integument (24 DAF)
Embryo
19 DAF
21 DAF
24 DAF
27 DAF
30 DAF
Outer integument (24 DAF)
Inner integument (21 DAF)
Embryo
35 DAF
40 DAF
43 DAF
46 DAF
49 DAF
Outer integument (30 DAF)

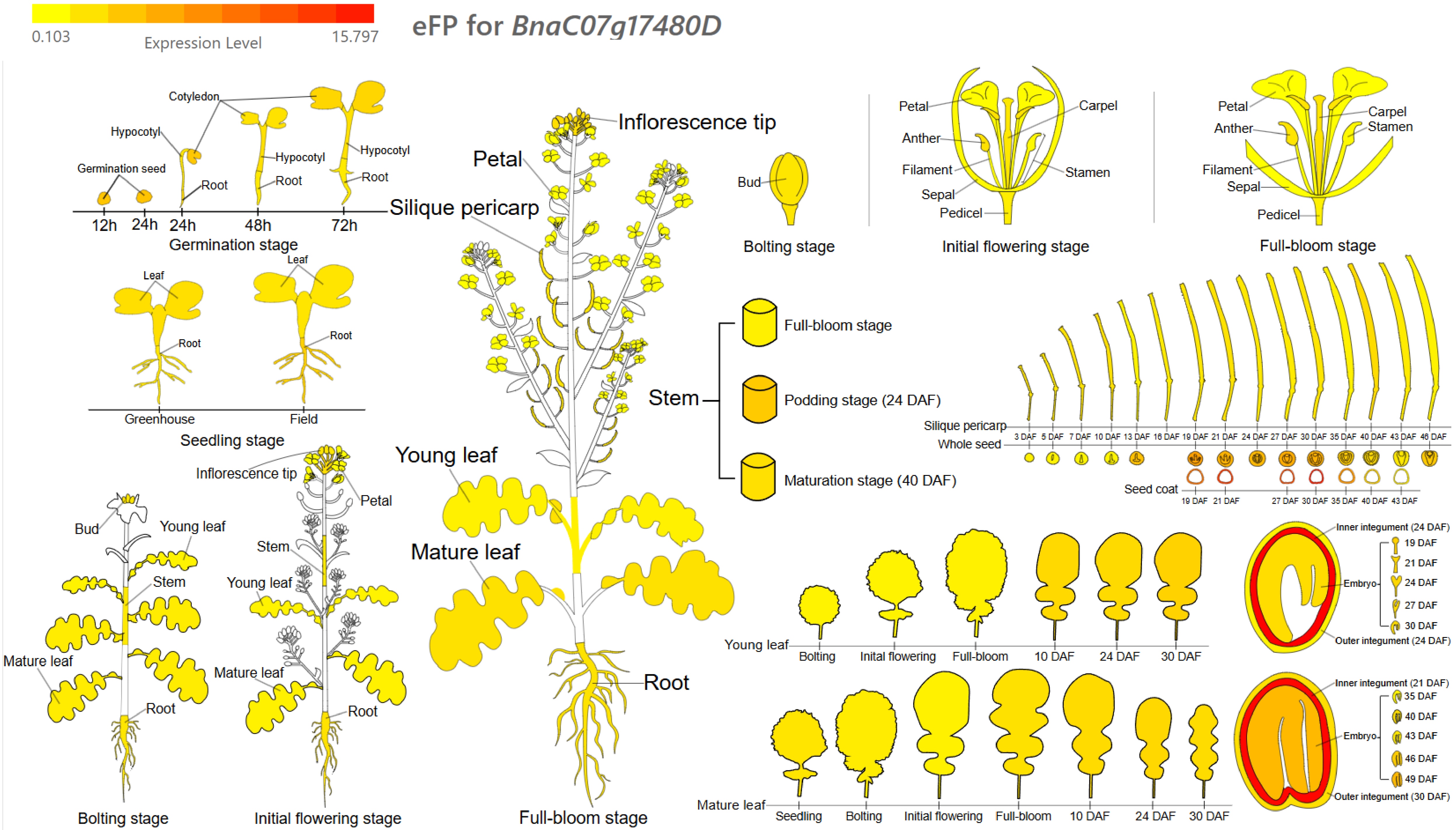

eFP for BnaC07g17480D
0.103
Expression Level
15.797
Cotyledon
Hypocotyl
Germination seed
Root
Hypocotyl
Root
Hypocotyl
Root
12h
24h
24h
48h
72h
Germination stage
Leaf
Root
Leaf
Root
Greenhouse
Field
Seedling stage
Inflorescence tip
Petal
Silique pericarp
Young leaf
Mature leaf
Root
Full-bloom stage
Bud
Young leaf
Stem
Mature leaf
Root
Bolting stage
Inflorescence tip
Petal
Stem
Young leaf
Mature leaf
Root
Initial flowering stage
Bud
Bolting stage
Petal
Anther
Filament
Sepal
Pedicel
Carpel
Stamen
Initial flowering stage
Petal
Anther
Filament
Sepal
Pedicel
Carpel
Stamen
Full-bloom stage
Stem
Full-bloom stage
Podding stage (24 DAF)
Maturation stage (40 DAF)
Silique pericarp
Whole seed
3 DAF
5 DAF
7 DAF
10 DAF
13 DAF
16 DAF
19 DAF
21 DAF
24 DAF
27 DAF
30 DAF
35 DAF
40 DAF
43 DAF
46 DAF
Seed coat
19 DAF
21 DAF
27 DAF
30 DAF
35 DAF
40 DAF
43 DAF
Young leaf
Bolting
Inital flowering
Full-bloom
10 DAF
24 DAF
30 DAF
Mature leaf
Seedling
Bolting
Initial flowering
Full-bloom
10 DAF
24 DAF
30 DAF
Inner integument (24 DAF)
Embryo
19 DAF
21 DAF
24 DAF
27 DAF
30 DAF
Outer integument (24 DAF)
Inner integument (21 DAF)
Embryo
35 DAF
40 DAF
43 DAF
46 DAF
49 DAF
Outer integument (30 DAF)

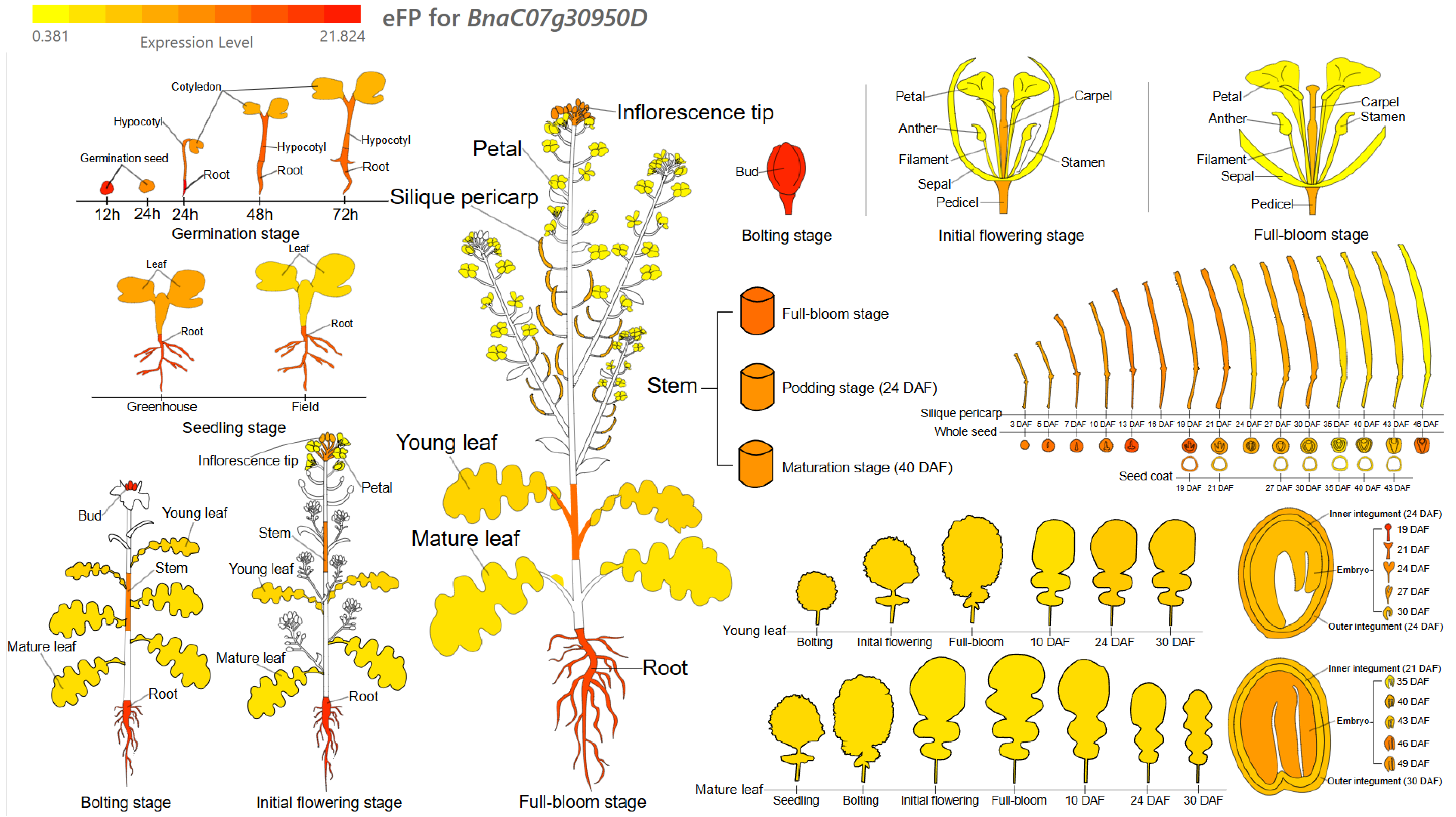

0.381
Expression Level
21.824
eFP for BnaC07g30950D
Cotyledon
Hypocotyl
Germination seed
Root
Hypocotyl
Root
Hypocotyl
Root
12h 24h 24h 48h 72h
Germination stage
Leaf
Leaf
Root
Root
Greenhouse
Field
Seedling stage
Inflorescence tip
Petal
Silique pericarp
Young leaf
Mature leaf
Root
Stem
Full-bloom stage
Inflorescence tip
Petal
Stem
Young leaf
Mature leaf
Root
Initial flowering stage
Bud
Young leaf
Stem
Mature leaf
Root
Bolting stage
Bud
Bolting stage
Petal
Anther
Filament
Sepal
Pedicel
Carpel
Stamen
Initial flowering stage
Petal
Anther
Filament
Sepal
Pedicel
Carpel
Stamen
Full-bloom stage
Full-bloom stage
Podding stage (24 DAF)
Maturation stage (40 DAF)
Silique pericarp
Whole seed
3 DAF 5 DAF 7 DAF 10 DAF 13 DAF 16 DAF 19 DAF 21 DAF 24 DAF 27 DAF 30 DAF 35 DAF 40 DAF 43 DAF 46 DAF
Seed coat
19 DAF 21 DAF 27 DAF 30 DAF 35 DAF 40 DAF 43 DAF
Young leaf
Bolting
Inital flowering
Full-bloom
10 DAF
24 DAF
30 DAF
Mature leaf
Seedling
Bolting
Initial flowering
Full-bloom
10 DAF
24 DAF
30 DAF
Inner integument (24 DAF)
19 DAF
21 DAF
24 DAF
27 DAF
30 DAF
Embryo
Outer integument (24 DAF)
Inner integument (21 DAF)
35 DAF
40 DAF
43 DAF
46 DAF
49 DAF
Embryo
Outer integument (30 DAF)
